# Acceptability and feasibility of a vestibular nerve stimulation headset protocol in children with cerebral palsy

**DOI:** 10.1186/s12887-021-03093-1

**Published:** 2022-01-11

**Authors:** Karen McConnell, Daniel Topley, Jason McKeown, Claire Kerr

**Affiliations:** 1 School of Health Sciences, Ulster University, Shore Road, Newtownabbey, BT37 0QB Northern Ireland UK; 2 School of Nursing and Midwifery, Queen’s University Belfast, Medical Biology Building, 97 Lisburn Road, Belfast, BT9 7BL Northern Ireland UK; 3 Neurovalens Ltd, 7 James Street South, Belfast, BT2 8DN Northern Ireland UK

**Keywords:** Cerebral palsy, Vestibular nerve stimulation, Balance

## Abstract

**Background:**

Research suggests electrical Vestibular Nerve Stimulation (VeNS) may improve balance for people with neurological impairments. This study aimed to assess the feasibility and acceptability of a VeNS headset protocol in children with cerebral palsy (CP).

**Methods:**

Children aged 5–18 years with ambulant CP, their parents, and healthcare professionals were recruited via social media. Children completed a battery of balance tests and wore a sham VeNS headset one hour per day for four weeks. Perspectives on the balance tests and headset were ascertained from children, parents and healthcare professionals using semi-structured interviews. Interview data were analysed thematically.

**Results:**

Two families and four healthcare professionals participated. Balance outcome measures were fully completed and deemed acceptable. Adherence with wearing the headset was 89–100% but discomfort with self-adhesive electrodes was reported. Four themes emerged from interview data: headset issues, perceptions about VeNS, the importance of balance, and modifications for future study.

**Conclusions:**

Although the VeNS headset had high acceptability, the volunteer sample was small, potentially suggesting limited interest in VeNS as a treatment for children with CP, or reluctance to trial a ‘non-active’ headset. Recruitment via clinicians known to the family and use of an ‘active’ headset may increase participation in future research.

**Supplementary Information:**

The online version contains supplementary material available at 10.1186/s12887-021-03093-1.

## Background

Cerebral palsy (CP) is a disorder of movement and posture that is caused by non-progressive disturbances to the developing foetal or infant brain [1]. Children with CP may experience decreased postural stability [2, 3], which may in turn lead to difficulties with gross motor skills [4, 5] and functional activities [2, 5, 6]. Stimulation of the vestibular system, which contributes to balance, may reduce balance deficits in people with CP. A recent systematic review [7] reported that 48% of children with spastic CP had vestibular dysfunction as measured through cervical vestibular evoked myogenic potential (cVEMP) [8] thus vestibular nerve stimulation interventions warrant investigation in people with CP.

Improvements in postural stability in children with CP have been demonstrated by stimulating the vestibular system via specific exercises [9–11]. However, a recent systematic review of the efficacy of vestibular stimulation in improving balance and function in people with CP highlighted contradictory results and methodological concerns in the underpinning literature [12]. All of the studies in the latter review evaluated vestibular stimulation in the form of exercise and movement: no studies were identified that evaluated electrical Vestibular Nerve Stimulation (VeNS) in CP. This is in spite of promising early research demonstrating improved postural stability with VeNS in patients with Parkinson’s disease [13–15], bilateral vestibulopathy [16] and the elderly population [17, 18]. VeNS is typically delivered via a headset that delivers electrical current via self-adhesive pads on the mastoid processes [19]. It can be conveniently delivered in the home environment [12], may be better tolerated than more traditional spinning and swinging exercises, and has the potential to impact on postural stability in children with CP.

Prior to developing large scale clinical evaluations on new interventions, feasibility studies are recommended to establish if, how and whether a future clinical trial should be conducted [20–22]. For example, it is important to understand if it is feasible to identify, recruit and retain children with CP for a VeNS intervention. In addition, consideration of service users’ perspectives of the acceptability of an intervention is necessary. Therefore, the current study aimed to assess the acceptability and feasibility of a VeNS headset protocol as a possible balance intervention for children with CP.

## Methods

### Study aims

The current feasibility study aimed to evaluate:

1. Rates of participant identification, recruitment and retention to the study;
2. Child/family adherence with wearing the VeNS headset;
3. Acceptability of the outcome measurements; and
4. Acceptability of a VeNS headset protocol as an intervention for children with CP from the perspectives of the child, family and healthcare professionals.

### Study design

A convergent parallel mixed methods approach was adopted. Quantitative data were used to address questions relating to recruitment and adherence, whereas qualitative data were used to help understand acceptability of the intervention and outcome measurements from the perspectives of children, parents and healthcare professionals.

### Participants and recruitment

Children with CP and their parents / guardians: We aimed to recruit 10 children with ambulant CP (Gross Motor Function Classification System (GMFCS) levels I-III [23]; aged five to 18 years in 2019, and their parent / guardian to the study. Data from the Northern Ireland Cerebral Palsy Register (NICPR) suggested there were approximately 560 children in the region that were potentially eligible for inclusion in the study [24]. Children with severe cognitive impairment (by parent report), and families who were unable to commit to the study period were not eligible to participate.

Healthcare professionals: We aimed to recruit five healthcare professionals involved in the clinical management of children with CP, who had an interest in balance.

Children and their parents / guardians were recruited via a flyer on social media platforms, including Facebook and Twitter over a three-month period (April-June 2019). The flyer was also emailed directly to subscribers of the NICPR community mailing list. Healthcare professionals were recruited via the same online methods, as well as the flyer being directly emailed to clinicians that inform the NICPR. Potential participants were asked to contact the research team directly to discuss their eligibility. A study information pack was sent to eligible families and healthcare professionals via post or email. Following a two-week ‘cool-down’ period, the research team re-contacted potential participants to answer questions and arrange the study visits. Informed consent was obtained from parents and healthcare professionals; assent was obtained from children. All participating families and healthcare professionals were given a £30 gift voucher as a token of appreciation for their time and participation in the study. Participants were not informed of this appreciation voucher until they had completed the study.

### Procedure

#### Children with CP and their parents / guardians

**Fig. 1:**
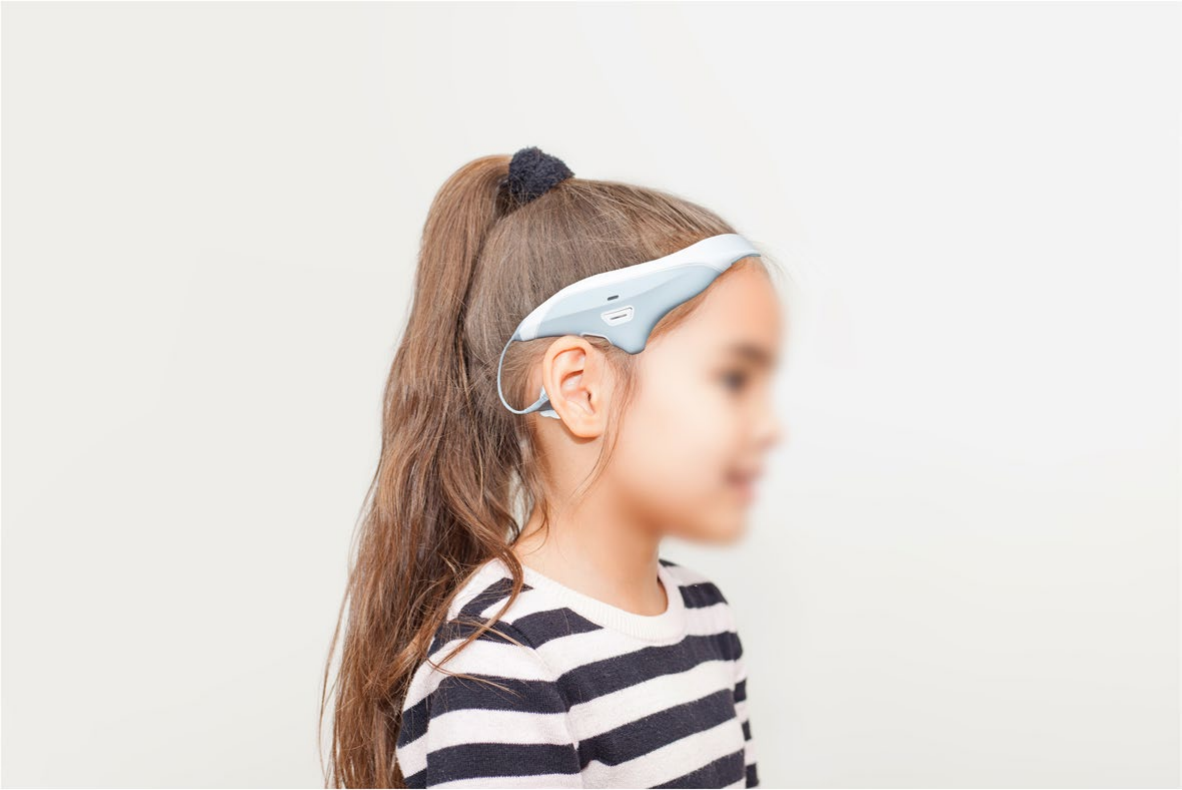
The VeNS headset used in this study

Children aged 5–18 years old with ambulant CP attended two appointments. At the first appointment the child completed the following balance measures at the Northern Ireland Clinical Research Facility with a trained research assistant (DT): Trunk Control Measurement Scale [25], Functional Reach Test [26], Paediatric Balance Scale [27], Timed Up and Go Test [28], Timed One-Leg Stance [29], Heel-To-Toe Stand [29] and One Minute Walk Test [30]. Following completion of the balance measures, the research assistant explained the application and care of the VeNS headset to the child and family. Families were also shown a short video demonstration of the headset. A sham VeNS headset (Modius MS600 headset by Neurovalens) with electrodes and cleaning wipes was then provided to the family. No active stimulation was delivered as the purpose was to ascertain comfort, fit and durability of the headset. The child was asked to wear the sham VeNS headset for one hour per day for four weeks whilst in a resting position (sitting or lying). During this headset trial, participants logged their use and any issues or concerns associated with wearing the headset on a daily basis in a paper diary. The VeNS headset employed is shown in Fig. 1.

At the end of the four-week period, the child and their parent attended a second appointment to complete one-to-one semi-structured interviews on the acceptability of the VeNS headset and outcome measurements used. Interview guides for children and parents were developed for the purposes of this study (Additional file 1). Interviews were conducted by a trained researcher and audio-recorded.

#### Healthcare professionals

Healthcare professionals were emailed study information packs. Verbal information about VeNS and the headset was provided by telephone (if requested) prior to completion of the interviews. The research assistant conducted individual semi-structured telephone interviews with each participating healthcare professional to discuss acceptability of using VeNS in children with CP. The interview guide for healthcare professionals was developed for the purposes of this study (Additional file 2).

### Data analysis

In line with a convergent parallel mixed methods design, quantitative and qualitative data were analysed separately and results subsequently integrated. Quantitative data on the feasibility of VeNS (Aims 1 and 2) were summarised using descriptive statistics. Quantitative data were analysed using Microsoft Excel. Qualitative interview data relating to the acceptability of balance outcome measures (Aim 3) and VeNS (Aim 4), were transcribed verbatim and analysed thematically according to the processes described by Braun and Clarke [31]. A deductive approach was used based on pre-defined research questions. One independent researcher (KMC) read and re-read transcripts and listened to the interviews to code qualitative data by hand. Investigator triangulation was used whereby subsequent themes were derived from coded data independently by two researchers (KMC and CK) to enhance credibility of the findings. Themes were then discussed and agreed by the researchers. Due to time constraints on the research, it was not possible to obtain feedback from participants on the qualitative analysis.

## Results

### Participants

Two children and their parents were recruited to the study. Both children had ambulant CP (GMFCS I-II), were male, and were aged five and 12 years old. Four healthcare professionals, all of whom were paediatric physiotherapists, were also recruited to the study.

### Study aim 1: Families—rates of participant identification, recruitment and retention

Identification: Of the 560 potentially eligible participants in the region, less than 3% (*n* = 15/560) responded to the recruitment flyer advertised via social media and a mailing list.

**Fig. 2:**
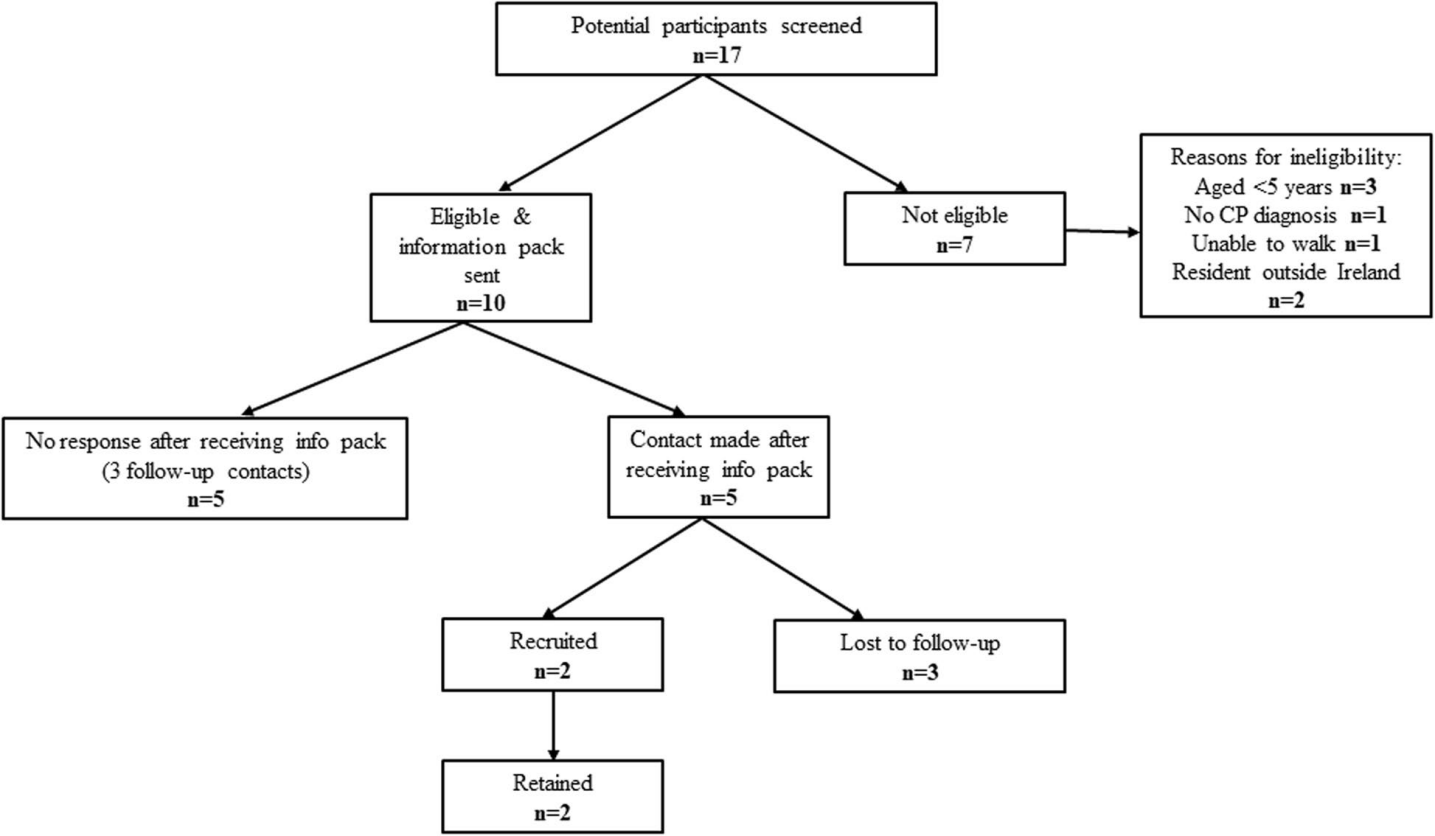
Flow diagram showing recruitment of children and their parents

Recruitment: The 15 families that responded during the three-month recruitment period were screened for eligibility. Of these, seven families were not eligible to participate for the following reasons: the child was aged < 5 years (*n* = 3), did not have CP (*n* = 1), was unable to walk (*n* = 1) or was resident outside the study area (*n* = 2). The eight eligible families were sent information packs. Three of those did not respond to further follow-up. Of the remaining five families, a further three were lost to follow-up. Thus only two families were recruited to the study (Fig. 2).

Retention: Both families participated in all aspects of the study.

### Study aim 1: Healthcare professionals—rates of participant identification, recruitment and retention

Identification and recruitment: Eight paediatric physiotherapists responded to the recruitment flyer, of whom two did not submit contact details. The remaining six received an information pack by email. Two physiotherapists were lost to follow-up after receiving the information pack, resulting in four being recruited to the study.

### Study aim 2: Adherence with a VeNS headset protocol

Examination of family-completed daily diaries showed that one participant wore the headset every day during the four-week study period, for an average of 53 min per day (range 30–75 min). The second participant wore the headset on 25 of 28 days for an average of 56 min per day (range 30–60 min).

### Study aim 3: Acceptability of the balance outcome measures

Both children completed all balance tests. The only item found to be challenging was item 13 of the Pediatric Balance Scale (placing alternate foot on a stool) [27], demonstrating a ceiling effect on this measure.

### Study aim 4: Acceptability of a VeNS headset protocol from the perspectives of children, families and healthcare professionals

Eight semi-structured interviews were conducted: two children and two parents completed face-to-face interviews and four paediatric physiotherapists participated in telephone interviews. Four broad themes emerged from the interview data: (i) headset issues, (ii) perceptions about VeNS, (iii) the importance of balance, and (iv) modifications for future study.

#### Headset issues

Children, parents and physiotherapists reported several positive and negative aspects to the VeNS headset employed in the study. Overall, participants found the headset aesthetically pleasing, with one therapist stating “it looks quite appealing, it looks quite cool”. However, one parent reported “if he was playing with his head down, he’d find that it would be drooping down on him.” Families also reported difficulties with the electrodes being “too sticky” and “itchy”. In addition, parents acknowledged their motivation was less as they knew their child was using a sham headset with one parent saying, “…if it had been connected to something, and there was an end result, it would have been different…”.

#### Perceptions about VeNS

All children and families reported that they would consider using VeNS to improve balance. Therapists were also largely supportive of using VeNS for balance with one stating, “I think it’s got great potential…I’d love to see it in practice.” Another therapist suggested that VeNS could be used as an adjunct therapy, “I can see it working well alongside – in conjunction with exercise” as opposed to an intervention delivered in isolation.

In terms of the VeNS protocol employed in this study, parents liked the flexibility of the child being able to wear the headset at different times each day, however one family “found it harder at the weekends, sometimes we’d forget…” In addition, children felt they had to “wear the headset too much” and for “too long”, and one parent agreed that, “… [one hour] seemed too long. At times he’d want it off. And then there were other days it’d pass quick enough.” However, families reported improved adherence when children were distracted whilst wearing the headset e.g. eating dinner, playing or doing homework.

#### The importance of balance

All paediatric physiotherapist participants acknowledged the importance of balance in the assessment and treatment of children with CP. One therapist stated that “[balance] certainly underpins a lot of our treatment focusses in regards to goals and the treatment programmes”, and another reported that balance is “one of the mainstays of our treatment, genuinely, for our kids who are mobilising and the higher level kids with CP, and balance definitely is a main focus of our treatment.” Similarly, one parent stated “for me [balance is] very important. Especially at his age, with going through school and things, and the things that he’s doing at school.” Whereas another family felt that balance was not a priority, “It’s more the non-use of the [right] side… his balance is pretty ok.”

#### Modifications for future study

Physiotherapists’ willingness to help with recruitment for future VeNS research was largely positive, but was dependent on organisational factors, such as their employer’s research policy and approval from their line manager, but also on the outcomes and experiences reported by children and families during this feasibility study, and the level of input required from families. One therapist reported: “I have sort of mentioned the topic to a few families already, and think that they would certainly be willing to have a go in the study…I’ve got patients around the UK…we’ve got quite a few physiotherapists working with us now, so I think it would be quite easy to get patients involved in a study that includes VeNS.”

All participants acknowledged that use of a mobile app in future VeNS research would be beneficial. Children felt the use of an app would be “cool” and a “good idea”, and a physiotherapist reported, “I am aware that kids, particularly that are old enough, do enjoy the feedback that would give them, and I do think that might improve compliance – especially if the app was able to reward them if they’ve done so many hours…”.

Whilst families felt the balance measures employed were appropriate, therapists made suggestions for additional outcome measures that may be useful in future studies. One therapist suggested inclusion of a quality of life measure and another stated that “…if you’re more steady then maybe you’ll need less effort to walk say, or stand still, and maybe oxygen consumption might be something you could look at, or heart rate.” In addition, two therapists suggested use of more discrete balance measurements that would require specialised equipment, or access to a gait analysis laboratory: “[the measures] may be just a wee bit too crude to pick up subtle improvements in balance, if you know what I mean. Whereas a pressure platform might do that for you.”

### Synthesis/Integration

Results from the quantitative and qualitative strands identified high acceptability of balance outcome measures and the VeNS headset however; the small sample size suggests that families and children with CP may have less motivation to prioritise a VeNS intervention over other therapy approaches.

## Discussion

This convergent, parallel, mixed methods study highlighted difficulties recruiting children with CP, their families and healthcare professionals to evaluate the feasibility and acceptability of VeNS. Families that participated in the study reported excellent adherence with wearing the sham VeNS headset, despite discomfort using the self-adhesive electrodes. Interview data identified four themes relating to the acceptability of a VeNS headset protocol in the CP population: (i) headset issues, (ii) perceptions about VeNS, (iii) the importance of balance, and (iv) modifications for future study. Overall, acceptability of the VeNS headset was high but the volunteer sample was small suggesting less interest in VeNS as a treatment option for CP.

Recruitment of potential children and families to the study was very low, with less than 3% of the potentially eligible population [24] responding to the recruitment flyer advertised via social media and a registry mailing list. This is in contrast to previous studies in our region where 38–47% of the potentially eligible CP population have been recruited [32, 33]. Interestingly, the latter studies used the NICPR to identify all eligible participants, who were then invited to participate by a clinician known to them. Due to the short time period and the small number of participants required for this study, a pragmatic recruitment approach using social media and an existing mailing list was used. Although the literature suggests recruitment via social media is cost-effective and can increase recruitment rates [34–36], it was not effective for this study. We suggest that future studies ensure sufficient time and resources are allocated to maximise participant recruitment via the use of population-based registers.

Another factor that may have impacted participation in the study was provision of a sham device – families may have chosen not to participate as no active intervention was being delivered. Finally, as CP is a complex condition that requires children to attend a range of healthcare services [37–39], it may be that participation in this research was considered to be an additional appointment burden on families.

Despite difficulties with recruitment, participating families reported excellent adherence to the VeNS headset protocol during the four-week trial period. This is in contrast to previous studies that involved home-based electrical stimulation interventions in children with CP [40, 41]. However, it is important to note that the current study recruited only two children and involved a sham device thus it is difficult to make comparisons with adherence rates reported in previous electrical stimulation research.

Overall the VeNS headset was accepted by children, parents and healthcare professionals in terms of aesthetics and comfort. Whilst children and parents reported disliking the self-adhesive electrodes that attached to the headset (see Fig. 1), this did not have an impact on reported adherence. Our results suggest that VeNS may be an acceptable treatment for balance in the CP population, but should be used in conjunction with exercise. Furthermore, prior to planning a clinical trial, use of an active VeNS headset should be explored to determine if children tolerate the sensation of the electrical stimulation.

The balance outcome measures in this study were selected based on evidence of validity and reliability in CP, age appropriateness of the tools, and type of balance being assessed [42, 43]. Our results suggest that children, their parents and paediatric physiotherapists considered the measures to be acceptable tools for assessing balance in children with ambulant CP. Paediatric physiotherapists suggested subtler changes in balance may only be detected more sensitive tools, such as pressure plates. In addition, it would be valuable to include measures of vestibular end-organ function to evaluate magnitude of effect in children with intact vestibular function versus those with vestibular dysfunction. However, such tools are not typically available in a clinical therapy environment and thus would incur additional equipment and staff training costs.

Families and healthcare professionals were positive regarding VeNS as an adjunct to more traditional therapy. However, the low volunteer sample suggests that only the most motivated families are likely to adopt the intervention. In addition, families of children with CP may not consider balance, or indeed VeNS, as a priority intervention considering the range of health and social care services currently used to manage the condition. Upon reflection, the authors acknowledge that it would have been useful to find out why those who were initially interested in the study did not take part.

### Strengths and limitations

Strengths included that data collection was completed by a trained research assistant and there were no deviations from the study protocol. Whilst, the sample size in this study was small, use of a convergent parallel mixed methods approach enabled fulfilment of important questions on recruitment methods, retention, adherence with a sham device and acceptability of balance outcome measures. In addition, the qualitative data provided useful feedback from families and healthcare professionals. Nonetheless, this study was limited by evaluating acceptability of using a sham VeNS headset and no active treatment was delivered.

## Conclusions

Recruitment via social media and online mailing lists resulted in a small sample size. Recruitment via a population-based register and healthcare professionals, in addition to use of an ‘active’ VeNS headset, may improve child and family participation in future VeNS research. Whilst adherence to the VeNS protocol was good, it is not possible to draw substantive conclusions in this regard due to the small number of participants recruited. The balance tests and headset employed were considered to be acceptable to families and healthcare professionals. In spite of this preliminary work, a larger clinical trial is not yet feasible due to the small sample size in this study and the lack of exploration of the sensation of VeNS. In addition, future research should use VeNS as an adjunct to conventional balance exercises.

## Supplementary Information

**Additional file 1.** Semi-structured interview schedules for children and parents/guardians

**Additional file 2.** Semi-structured interview schedule for healthcare professionals

## Data Availability

The datasets used and/or analysed during the current study are available from the corresponding author on reasonable request.

## Abbreviations

CP: Cerebral palsy
VeNS: Vestibular nerve stimulation
