# Supplementary material for: Acceptability and feasibility of a vestibular nerve stimulation headset protocol in children with cerebral palsy
Source: BMC Pediatr. 2022 Jan 11;22:34. doi: 10.1186/s12887-021-03093-1 (PMC8750849; doi:10.1186/s12887-021-03093-1)
Supplement: Supplementary file 1 — Additional file 1. Semi-structured interview schedules for children and parents/guardians [file 12887_2021_3093_MOESM1_ESM.pdf]

Additional file 1: Semi-structured interview schedules for children and parents/guardians

Semi-structured interview schedule for children

1. Thank the participant and read:

"I'd like to ask you some questions to find out what you think about the headset you wore at home for four weeks and the tests we asked you to do for your balance. I'd like to record the interview so I can listen back to it afterwards, but everything you tell me will be private, and will only be shared by the researchers. I would like to hear your own opinion, and so there are no right or wrong answers. It doesn't matter if what you say is positive or negative. And finally, you may stop the interview at any time without giving a reason."

2. Obtain verbal consent (written consent obtained at start of study).

3. Demonstrate the app that is used alongside an active VeNS headset.

4. Test recording device.

5. Start recording device and conduct interview using example questions below. Start the recording by saying:

"This interview is with [participant name] on [date and time]."

Example questions for interview (age dependent):

<u>*About the headset*</u>

- What did you think about the headset you wore for 4 weeks?

  Probes:

  - What was it like to wear the headset?
  - How did you find the sticky pads?
  - What did you like about the headset?

    - Was there anything you did not like about the headset?
    - Would you change anything about the headset?

- What time of the day did you prefer to wear the headset?
- What were you doing when wearing the headset?

    Probes:

    - Sitting / lying down?

- Did wearing the headset interfere with your normal activities?
- What do you think about low long and how often we asked you to wear the headset?

    Probes:

    - Were you happy to wear the headset for a full hour each time?
    - Do you think you wore the headset too much / too little?

- What do you think about the mobile phone app that is used with a 'real' headset?

<u>About balance</u>

- What do you think about your balance?

    Probes:

    - Is balance important to you?
    - Would you like to improve your balance? Why / Why not?

- What did you think about the tests / exercises you did to find out about your balance?

    Probes:

    - What did you like about the balance tests?
    - Was there anything you did not like about the balance tests?
    - Would you change anything about the balance tests?

- Would you like to use the VeNS headset to help your balance? Why / why not?

About future VeNS research

- Would you like to do more research using the VeNS headset? Why / why not?
- What would you think about the headset sending electrical messages to your brain?
- In future research there would be two groups – one group would receive an 'active' VeNS headset that delivers stimulation, the second group would receive a 'practice' headset like the one in this study. If you decided to do more research to find out if the headset works, would you be happy to get another 'practice' headset instead of a real one? Why / why not?

6. At the end of the interview read:

"This is the end of the interview with [participant name]."

7. Stop recording device and thank participant.

Semi-structured interview schedule for parents / guardians

1. Thank the participant and read:

"I'd like to ask you some questions to find out what you think about the headset your child wore for four weeks and the tests we asked him / her to do for their balance. I'd like to record the interview so I can listen back to it afterwards, but everything you tell me will be confidential, and will only be shared by the research team. I would like to hear your own opinion, and so there are no right or wrong answers. It doesn't matter if what you say is positive or negative. And finally, you may stop the interview at any time without giving a reason."

2. Obtain verbal consent (written consent obtained at start of study).

3. Demonstrate the mobile phone app that is used alongside an active VeNS headset.

4. Test recording device.

5. Start recording device and conduct interview using example questions below. Start the recording by saying:

"This interview is with [participant name] on [date and time]."

Example questions for interview:

<u>*About the headset*</u>

- What did you think about the headset your child wore for 4 weeks?

    Probes:

    - How did your child find wearing the headset?
    - How did you / your child find the electrodes (sticky pads)?
    - What did you / your child like about the headset?
    - Was there anything you / your child did not like about the headset?

    - Would you change anything about the headset?
- What time of the day did your child prefer to wear the headset?
- What did your child do when wearing the headset? E.g. resting

    Probes:

    - Did wearing the headset interfere with your child's / family's normal activities?
- What did you think about how long and how often we asked your child to wear the headset?

    Probes:

    - Was your child happy to wear the headset for a full hour each time?
    - Do you think he / she wore the headset too much / too little?
- What do you think about the mobile phone app that is used with an active VeNS headset?

<u>*About balance*</u>

- What do you think about balance / your child's balance?

    Probes:

    - Is it important to treat your child's balance?
    - Would you like your child's balance to improve? Why / why not?
- What did you think about the tests / exercises your child did to find out about their balance?

    Probes:

    - Did you /your child like about the balance tests?
    - Was there anything you / your child did not like about the balance tests?
    - Would you change anything about the balance tests?
- Would you like your child to use the headset if it helps their balance? Why / why not?

About future VeNS research

- Would you like your child to take part in more research using this headset to find out if it helps their balance? Why / why not?
- What would you think about your child using the headset when it is switched on / provides electrical stimulation?
- In future research there would be two groups – one receiving an 'active' VeNS headset and one receiving a 'practice' headset like the one in this study. If your child did take part in more research to find out if the headset works, would you be happy if they were put into a group that received another 'practice' headset without stimulation?

6. At the end of the interview read:

"This is the end of the interview with [participant name]."

7. Stop recording device and thank participant.
