# Supplementary material for: Acceptability and feasibility of a vestibular nerve stimulation headset protocol in children with cerebral palsy
Source: BMC Pediatr. 2022 Jan 11;22:34. doi: 10.1186/s12887-021-03093-1 (PMC8750849; doi:10.1186/s12887-021-03093-1)
Supplement: Supplementary file 2 — Additional file 2. Semi-structured interview schedule for healthcare professionals [file 12887_2021_3093_MOESM2_ESM.pdf]

**Additional file 2: Semi-structured interview schedule for healthcare professionals**

**1. Answer questions about the study and obtain written consent as participants arrive.**

**2. Introduction to VeNS, the VeNS headset and the potential application to improve balance in children with cerebral palsy:** This will involve (i) demonstration of the VeNS headset, how it is applied / used / cared for – allow some time for clinicians to handle the device and try it on if they wish, (ii) demonstration of the app that is used with an 'active' VeNS headset, and (iii) summary of how VeNS might affect balance in children with cerebral palsy.

**3. Thank the participants and read:**

"Thank you for joining us for this interview. I'd like to ask some questions to facilitate a discussion around the potential use of Vestibular Nerve Stimulation (VeNS) in children with cerebral palsy. I'd like to find out what you as healthcare professionals think about this new treatment. There may be different opinions in the group so I would ask that you respect others and give everyone a chance to share their thoughts. There are no right or wrong answers and it doesn't matter if what you say is positive or negative. I'd like to record the interview so I can listen back to it afterwards, but everything you say will be confidential, and will only be shared by the research team. And finally, you may stop / leave the interview at any time without giving a reason."

**4. Test recording device.**

**5. Start recording device and facilitate the interview using the example questions below.**

**Start the recording by saying:**

"This interview is with [participant names] on [date and time]."

**Example questions for interview:**

*About the headset*

- What do you think about the VeNS headset?
- What do you think about the app used in conjunction with the headset?

*About balance*

- What do you think about management of balance in children with CP?

  *Probes:*

  - Is it important?
  - How do you assess / treat balance in children with CP?
- What do you think about the measurement tools we used to assess balance in this study?

  *Probes:*

  - Are there any additional balance tests you would include? Why / why not?
- Do you think VeNS has the potential to improve balance in children with CP?
- What measurement tools do you think should be used to evaluate the efficacy of VeNS in children with CP?

*About future VeNS research*

- What would you think about some of your patients taking part in research to evaluate the efficacy of VeNS in children with CP?
- Would you support recruitment of children with CP to a large scale clinical trial evaluating VeNS?
- Would you be interested in sitting on an Advisory Group to help plan future research on VeNS in children with CP?

**6. At the end of the interview group read:**

"This is the end of the healthcare professional's interview."

**7. Stop recording device and thank participants.**
